# Environmental Measurement and Control System for Animal Health Research Using Arduino †

**DOI:** 10.3390/s26010053

**Published:** 2025-12-20

**Authors:** Dan Hofstetter, Serenity M. Wilcox, Ruijie Wang, Eileen E. Fabian, Alberto Gino Lorenzoni

**Affiliations:** 1 Department of Agricultural and Biological Engineering, University of Florida, Gainesville, FL 32611, USA; serenitywilcox@ufl.edu (S.M.W.); ruijiewang@ufl.edu (R.W.); 2 Department of Agricultural and Biological Engineering, The Pennsylvania State University, University Park, PA 16802, USA; 3 Department of Animal Science, The Pennsylvania State University, University Park, PA 16802, USA; agl20@psu.edu

**Keywords:** Arduino, data acquisition, ammonia control, dust level, indoor environment, sensor array, poultry

## Abstract

Recent advances in electronics components such as microcontrollers and sensors, plus widespread availability of 3D printing, make it possible to construct all-in-one datalogger and control systems in purpose-built enclosures. In addition, microcontrollers can be easily reprogrammed for different purposes including environmental measurement and control of external devices. Since specific needs vary widely across agricultural systems, on-farm research often requires custom instrumentation and datalogging solutions. A custom-built all-in-one environmental measurement and control system (EMCS) was developed for animal health research studies. The system integrates an array of environmental sensors to detect and record luminosity, carbon dioxide and ammonia gas concentrations, dust particle counts, air speed, temperature, and humidity. The developed system is presented along with a case study where environment monitors were deployed to control ammonia and dust in controlled environment chambers for a six-week broiler health study.

## 1. Introduction

It is common for farmers and researchers to visit animal barns on a regular basis. However, if a problem is not noticed during routine inspection, it may go undetected for an extended duration. Dataloggers with environmental sensor arrays are extremely useful for measuring and quantifying indoor living conditions for animals raised in barns. Having accurate logs of environmental parameters is useful for recordkeeping and allows for analysis of effects of the indoor environment on animal performance and welfare. In addition to being important for farm operations, environmental measurement and control systems are a necessity for farm animal research. Indoor temperature [1,2], relative humidity [3], indoor air quality [1,4], and lighting conditions [5] affect the health and welfare of farm animals raised indoors and can all be monitored and recorded to document conditions in which animals are being raised. Comparison of current measurements with previously recorded ones can help to predict challenges related to extreme weather conditions and climate change [6,7,8], and it can help identify issues before they translate into animal disease.

It is not uncommon to use multiple instruments to record measurements during research studies in animal barns. Each instrument may come with its own software required to access instrument data logs, or the instrument may have built-in storage accessible via a USB connection or a storage card. Saving and merging data files from multiple instrument data logs can be a tedious task, particularly when the data formats do not match, internal clocks are not perfectly synchronized, or logging intervals are different. All of this adds up to a significant time requirement to gather, organize, and merge instrument data logs if analysis needs to be performed on the whole data set.

An environment measurement and control system (EMCS) was needed for an animal health research study to evaluate the physiological effects of exposure to controlled levels of ammonia gas and airborne dust [4]. Commercially available dataloggers were considered which could be programmed to turn external devices on based on sensor readings, but an all-in-one solution that was easier to deploy and use was desired. Previous work by the authors demonstrated the feasibility of using an Arduino microcontroller to measure ambient indoor conditions and control connected devices based on real-time sensor feedback [9]. This article describes an environment measurement system that was custom-built for animal housing environments that also has the capability to be used as a controller for research instruments and equipment. It includes a discussion of hardware selection and configuration, software integration, and lessons learned from deployment in four different controlled environment chambers during a six-week broiler health study.

### 1.1. Evolution of Datalogger Use in Animal Barns

Instrumentation and datalogging systems have been widely used for research in animal barns to monitor indoor air quality, thermal comfort parameters, and pollutant emissions. Typical measurements include contaminant gas levels, airborne particulate matter, temperature, relative humidity, and air movement or air exchange rates. Since the early 2000s, datalogger technology has evolved from highly specialized and expensive systems to affordable, customizable, and user-friendly platforms.

Early systems such as ones described by Wilhelm et al. [10] used a Campbell Scientific CR21X datalogger with a multiplexer to record measurements from several electrochemical ammonia gas sensors in addition to temperature and relative humidity. A personal computer communicated with the datalogger over phone lines using a 1200 baud modem. One initial weakness of the system was that the datalogger could lose all recorded data if a power failure occurred. To overcome this problem, they used a newer model CR23X datalogger with non-volatile memory. Despite improvements, the total cost of the datalogging system was approximately $14,000, making it impractical for widespread use.

Subsequent work focused on developing portable solutions. Gates et al. [11] developed a portable monitoring unit (PMU) for measuring ammonia gas emissions from poultry houses that contained two electrochemical ammonia gas sensors with built-in dataloggers capable of storing 7200 data points. While portable, this system required an air sampling pump to draw barn air into the unit, and periodic purging with clean air to prevent sensor degradation. This limited its ability to perform continuous monitoring. The authors also noted the importance of calibration and signal conditioning to account for long-term drift of the electrochemical sensors after repeated exposure to ammonia gas.

Around the same period, Heber et al. [12] developed an on-site computer system (OSCS) that utilized a PC running LabView software (National Instruments, Austin, TX, USA) with a data acquisition card connected to an array of external sensors. Remote access to the PC was possible using third party software, allowing continuous monitoring and automation of connected devices. They emphasized the importance of logging measurements from multiple sensors to a single data file with the same time stamp to avoid errors when processing data from standalone devices.

The next critical evolution in the field emerged with the availability of low-cost microcontrollers. Abraham et al. [13] developed a low-cost indoor air quality monitoring system that used an Arduino microcontroller with wireless Xbee communication modules. The system was used to transmit six air quality parameters from multiple different locations. Using affordable sensors such as the DHT-22 for temperature and relative humidity and the MQ-7 to measure carbon dioxide gas concentration, the system demonstrated comparable performance to professional-grade devices at a fraction of the cost. Similarly, Beddows et al. [14] emphasized the ease of building and modifying Arduino-based systems, showcasing the flexibility and accessibility of modern prototyping platforms. Building on these advances, Ji et al. [15] upgraded the PMU concept using an Arduino MEGA 2560 microprocessor with an electrochemical ammonia gas sensor. The upgraded unit measured temperature, ammonia and carbon dioxide gas concentrations, and could be custom programmed for different monitoring schemes and was called the Intelligent PMU (iPMU). The total cost of the system was $2454 per unit. While the slow response time and need for purging the electrochemical ammonia gas sensors remained limitations, the system showed how microcontroller-based designs could combine portability, connectivity, and affordability. Dotto et al. [16] added a particulate matter (PM_2.5_) sensor and further renovated the iPMU in 2022 into an Internet of Things (IoT) system that could be remotely controlled over the cloud and included a web dashboard for data visualization and cloud-based data analysis. This progression from commercial dataloggers to flexible microcontroller systems paved the way for custom-built dataloggers to be tailored to specific needs.

### 1.2. Device Development Considerations

#### 1.2.1. Enclosure

Since custom dataloggers are application-specific, suitable pre-made enclosures may be difficult to find. Much of the time spent developing custom dataloggers is devoted to the enclosure [17]. Enclosures need to be rugged enough for the intended environment, and careful consideration is needed to make sure electronic components are adequately protected from water and dust intrusion [18]. Acrylonitrile Butadiene Styrene (ABS) plastic parts are very durable, but even with 100% infill, 3D-printed ABS plastic is still somewhat porous and not completely watertight. ABS plastic components can be treated after 3D printing to seal against moisture if required [19].

Modular housing designs that allow for easy expansion are desirable. If sensors are expected to require maintenance, it is important that enclosures and wiring connectors be designed for ease of sensor access and replacement. 3D printing increases versatility and enables easy changes or substitutions since simple adapters can be designed to plug openings or mount different parts in the same space versus having to print a new enclosure.

#### 1.2.2. Component Location

Temperature sensors may return false high measurements if the sensors are mounted too close to other electronic components that generate heat, such as voltage regulators and metallic oxide semiconductor (MOS) gas sensors with heated sensing elements. Deployment of the environment monitor is another important consideration [18]. Animals may damage hanging wires or sensors, so components should be hung out of reach or protected by enclosing them behind barriers. Wheeler et al. [17] recommended guidance for sensor locations and protective measures in different types of animal housing. Electrical supply considerations and surge protection with uninterruptible power supply (UPS) backup might also be important for plugged-in devices.

### 1.3. Microcontroller Considerations

Microcontrollers are available in many different types, with some of the most common based on the Arduino or ESP32. Considerations for use include size of the microcontroller, processor type and speed, programming memory, types of included interfaces for device interconnection, voltage requirements and power supply limitations, and number of analog or digital input/output (IO) pins.

The Inter-Integrated Circuit (IIC or I2C) bus developed by Philips Semiconductors [20] is one of the most common synchronous serial communications protocols in use today [21]. It utilizes a two-wire interface and allows multiple devices with different addresses to be connected to the same bus. Commonly, the clock (SCL) and data (SDA) pins on an Arduino or ESP32 microcontroller. However, device connection limitations occur if two devices with the same hardware address are connected to the same communications bus.

Software conflicts may also arise, and some sensor libraries cause conflicts when trying to communicate with multiple different components connected to the same microcontroller. Therefore, it is suggested that development be performed in steps: add one device or sensor, test, then add the next one when the unit is working as intended [18]. Incremental testing is important to ensure connecting an additional sensor does not affect operation of already working ones.

#### Communications

In the past, many dataloggers were deployed in remote locations where communication was not possible unless they were connected to a laptop computer. Recent advances have made it easy to incorporate wired or wireless communications. Advantages of a communications connection include being able to remotely monitor conditions in real-time, as well as the ability to download data files and make programming changes. Incorporation of both wired and wireless communication capabilities can help to ensure compatibility with a variety of networks. Cellular communication modules are also available if network hardware is not accessible.

### 1.4. Sensor Considerations

It is very important to consider the environment where instruments will be deployed when selecting gas sensors for an intended use, because gas sensors often have cross-sensitivity to more than one type of gas. An example of this would be selecting an MQ-135 sensor to measure carbon dioxide or ammonia gas concentration. Because the MQ-135 is sensitive to both carbon dioxide and ammonia [22,23], this would be a poor choice for poultry barns and other farm environments where both gases could be present. Some sensors are also cross-sensitive to carbon monoxide, which may be present in low concentrations in barns using gas heaters during winter [24].

The ideal sensor technology would be one that requires little to no maintenance or adjustment during use, but sensors should be easy to access to allow routine cleaning of accumulated dust and debris, and for replacement if necessary. This may be accomplished using connectors that plug in to a prototyping shield, or by designing ease of access into custom designed enclosures. For example, frequently changed sensors can be inserted into slots and secured in place using snap hooks if custom 3D-printed enclosures are used. The sensor should also be capable of continuous use in harsh environments containing elevated levels of particulate matter, which can settle and accumulate inside tiny flow passages, and protected from oxidation and corrosion that could degrade electrical connections or shorten the life of electronic components [25].

Sensor response time is also important, since some sensors return measured values more quickly than others, so microcontroller code execution times are often limited to the slowest connected components. And some sensors and microcontrollers may operate at different voltages, requiring logic level voltage conversion to avoid damage to components.

Sensors need to be carefully tested and calibrated to ensure measurements are accurate enough for the intended use. Periodic calibration checks can be performed to account for sensor aging or drift using other more recently calibrated sensors or reference instruments [18]. Despite being inexpensive, available, and easy to use, issues can arise if users do not understand the need for sensor calibration prior to and during use. Chojer et al. [26] reviewed recent developments of custom low-cost indoor air quality monitors. They mentioned the idea of “grey literature” where low-cost sensors become used for research, but may not be calibrated or do not detail validation against trusted instruments.

#### 1.4.1. Ammonia Gas Concentration Measurement

Lin et al. [27] developed a metallic oxide semiconductor (MOS) based ammonia monitor for poultry houses based on the Figaro TGS2444 sensor. The sensor exhibited a quicker step response to changes in ammonia gas levels compared to electrochemical sensors. They evaluated effects of air flowing over the sensor element on sensor response, hysteresis, and short-term drift, and demonstrated how temperature and humidity effects could be corrected. MOS gas sensors have also been used in electronic nose detectors to monitor indoor air quality parameters [28]. MOS sensors respond quickly to changes in gas concentration [29], but do not become consumed when continuously exposed to ammonia gas like electrochemical sensors [30], making them potentially suitable for continuous measurement in animal barns.

#### 1.4.2. Dust Concentration Measurement

Many commonly available optical particle counters are based on the principle of light scattering [31], where airborne particulate matter passes through a beam of light in a chamber, and a detector intercepts light scattered by particulates and calculated particle concentration is proportional to the amount of scattered light. The Plantower PMS5003 sensor (Plantower, Beijing, China) is an optical particle counter that uses a laser diode as a light source. Li et al. [31] used the PMS5003 laser particle counter to measure PM2.5 and PM10 dust concentrations in commercial poultry houses in China for three months, and comparisons to higher cost instruments have reported the PMS5003 sensor has good build quality, low limits of detection, and quick measurement times needed for continuous real-time monitoring [32,33].

## 2. Materials and Methods

**Figure 1:**
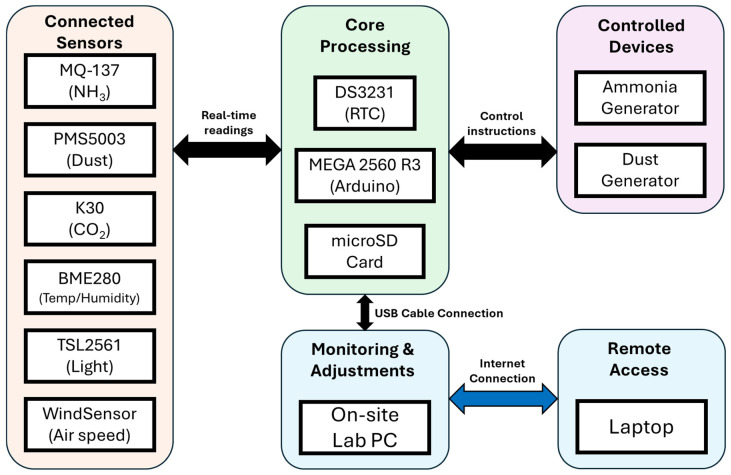
General block diagram of the EMCS with connected components and devices.

The custom environment measurement and control system (EMCS) described in this paper was developed incrementally in steps. First, datalogging capability was added to the microcontroller by connecting a real-time clock (RTC) and a microSD card storage module. Sensors were then connected one at a time to check for and troubleshoot any hardware or software conflicts from additional components or software libraries. After all components were integrated, testing was performed to evaluate sensor accuracy, response to measured variables, and control of connected devices under the same conditions under which the system would be deployed. Finally, four units were deployed and operated for a six-week poultry health study. Table A1 is a list of parts and electronic components used to build the environment measurement and control system. Figure 1 is a general block diagram of the EMCS that illustrates all connected sensors and devices described in this section.

### 2.1. Microcontroller

The EMCS was developed around an Arduino MEGA 2560 R3 microcontroller with 256 KB of flash memory (Elegoo, Inc., Shenzhen, China). It was prototyped using breadboards by adding one component at a time, then adding new code for each additional connected sensor and testing. Once everything worked well together, components were securely connected to the Arduino using wires and headers soldered onto a prototyping shield. Using a shield also allows for easy replacement of the microcontroller if needed.

### 2.2. Enclosure

**Figure 2:**
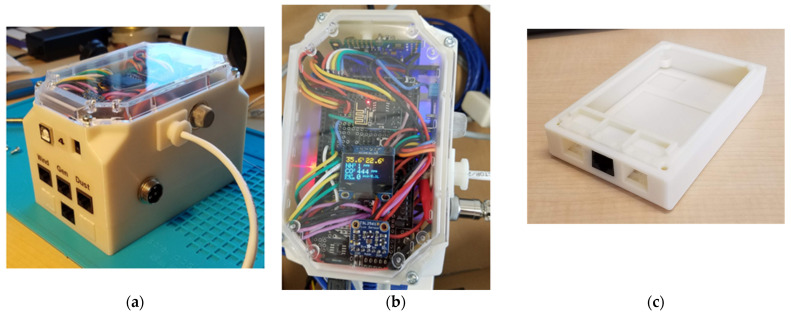
The developed environment measurement and control system assembled into a 3D-printed enclosure, (**a**) with one expansion spacer installed, (**b**) top view, (**c**) expansion spacer with bottom lid attached.

A clear polycarbonate enclosure lid was used for the enclosure. A custom housing was designed around the microcontroller and electronic components using SolidWorks 2015 (Dassault Systèmes SolidWorks Corporation, Waltham, MA, USA), and it was 3D-printed using ABS plastic with solid infill for durability. The bottom lid of the enclosure had a sliding door to allow access to the microSD card for data retrieval. Thread-forming screws were used to mount devices to the inside walls of the enclosure and to secure the top and the bottom lids. The 3D-printed housing was designed to be modular so additional sections for input or control could be added, with spaces for external device connections using RJ45 jacks that were held in place by the bottom lid. The main enclosure had extra space for batteries for future handheld use. The assembled enclosure had dimensions of 137 mm long × 89 mm wide × 79 mm high (5.4 in. × 3.5 in. × 3.1 in.). A 25 mm tall expansion spacer was also designed that could be mounted between the main enclosure and the bottom lid, providing space for up to three additional RJ45 jacks for connection to more external devices. Figure 2 shows an assembled EMCS in the 3D-printed enclosure with one expansion spacer installed.

### 2.3. Sensor and Component Selection

The EMCS was designed to continuously measure and record variables important to indoor animal production. The measured variables included luminosity, carbon dioxide (CO_2_) gas concentration, ammonia (NH_3_) gas concentration, temperature, relative humidity, air speed, and airborne dust particle counts. This section describes the sensors and components used in the EMCS and the reasons why they were selected.

#### 2.3.1. Luminosity Sensor

Luminosity (lux) was measured using a TSL2561 lux sensor (Adafruit Industries, New York, NY, USA). The sensor approximates human eye response and contains both infrared and full-spectrum photodiodes to measure from 0 to 40,000+ Lux. The sensor used 5 V power and had an I2C interface to communicate with the microcontroller.

#### 2.3.2. Carbon Dioxide Gas Sensor

Carbon dioxide concentration was measured using a nondispersive infrared (NDIR) K30 1% CO_2_ sensor (CO2Meter.com, Ormond Beach, FL, USA). Software serial communication via the K30serial library was used to send readings from the sensor to the microcontroller. The K30 CO_2_ sensor shipped with an automatic baseline correction (ABC) algorithm enabled by default, which constantly tracks the lowest sensor reading over a period of 7.5 days and adjusts for long-term drift compared to an expected CO_2_ concentration of 400 ppm in fresh air. To avoid calibration issues, we disabled ABC when using the K30 sensor in the environmental chambers housing broiler chickens because the expected level of CO_2_ would be always greater than 400 ppm. To disable ABC, the K30 sensor was connected to a PC running GasLab 2.0 software (CO2Meter.com, Ormond Beach, FL, USA) using a FTDI (USB to Serial) converter cable.

#### 2.3.3. Ammonia Gas Sensor

A metallic oxide semiconductor (MOS) ammonia sensor (MQ-137, Sainsmart, Lenexa, KY, USA) measured ammonia gas concentration. The MQ-137 sensor had an ammonia gas (NH_3_) detection range from 5 to 500 ppm and was not cross-sensitive to other gases expected to be present in the controlled environmental chambers. The sensor datasheet [34] recommended a 47 kΩ load resistor (R_L_) value, so the 10 kΩ resistor on the MQ-137 circuit board was removed and a 47 kΩ load resistor was soldered onto the prototyping shield between analog pin A2 and GND.

The MQ-137 ammonia gas sensors were tested for accuracy and calibrated in a controlled environment chamber at the upper span of concentration for our study. All four EMCS units were placed in the chamber in the same location as the sampling tube connected to a highly accurate Fourier Transfer Infrared (FTIR) reference instrument (model 7000 FTIR, California Analytical Instruments, Orange, CA, USA) and exposed to 65 ppm NH_3_ gas under steady temperature and humidity conditions fox six hours. The average MQ-137 gas sensor accuracy was within 1.4% of the 65.4 ppm concentration measured using [35].

#### 2.3.4. Temperature and Relative Humidity

Temperature and relative humidity were measured using a BME280 sensor module. The BME280 sensor (Bosch Sensortec, Reutlingen, Germany) could measure temperature from −40 to 85 °C (± 0.5 °C) with a resolution of 0.01 °C and humidity from 0 to 100% RH (± 3%) with a resolution of 0.008% RH [36]. The sensor was connected to 5 V power and the I2C bus on the microcontroller using a 203.2 mm (8 in.) long instrument cable and enclosed in a small external 3D-printed housing so the temperature reading would not be affected by heat from other electrical components inside the main EMCS enclosure.

#### 2.3.5. Real-Time Clock

A highly accurate DS3231SN real-time clock (RTC) module (Diymore, Shenzhen, China) was used to generate time and date for datalogging purposes. The DS3231SN module was selected because it integrated a 32 kHz temperature-compensated crystal oscillator (TXCO) and had an accuracy of ±2 ppm [37], or approximately ±63 s per year. The RTC module used 5 V power and communicated with the microcontroller using the I2C bus. The RTC module included a charging circuit which necessitated the use of rechargeable LIR2032 batteries.

#### 2.3.6. Data Storage

A microSD card module was used with a 16 GB microSD card (Patriot Memory, Fremont, CA, USA) to store logged data. The microSD card module had an SPI interface and was connected to the Arduino MEGA using pins 50, 51, 52, 53 (MISO, MOSI, SCK, SS). The microSD card was formatted using a FAT32 file system. All measured data were logged to the microSD card using a comma-separated value (.CSV) file format that could be easily opened using Microsoft Excel. A new file was created each day automatically at midnight with the filename YYYYMMDD.CSV (YYYY = year, MM = month, DD = day). One row of data was logged every 5 to 6 s, so each data file for a 24-h period contained approximately 15,000 rows and 29 columns of data and was approximately 2.1 MB in size. The first three rows of the data file contained the EMCS identifier and deployment details, the program location and file name, and the compilation date, which was useful for tracking program changes. The next line of data contained a string of column headings for each session. Table 1 lists the measured variables that were printed to the serial monitor window and logged to the microSD card.

#### 2.3.7. Display

A 24.4 mm (0.96 in.) 128 × 64 pixel yellow/blue OLED SSD1306 display module (Waveshare, Shenzhen, China) with I2C serial communications was used to show measured temperature, humidity, and ammonia, carbon dioxide, and dust concentration values locally at the EMCS. The display was powered using 5 V and connected to the microcontroller using the I2C bus. The display module was plugged in to a 4 × 1 female header soldered to the prototyping shield so that it could easily be replaced if needed.

#### 2.3.8. Wi-Fi

An ESP8266 ESP-01S Wi-Fi module (HiLetGo, Shenzhen, China) was connected to hardware serial port 2 (pins Tx2,Rx2) to enable data to be sent wirelessly for real-time online monitoring of environmental conditions. The Wi-Fi module was not used, but was tested and included for the future ability to check sensor readings via a smartphone app (such as telnet serial terminal) when walking through a barn.

#### 2.3.9. Internal Wiring and Connectors

Silicone wire (60 strands, 22-gauge) was used for all wiring inside the datalogger and other sensor housings because it is very flexible, making wire breakage less likely during assembly or use. Wires were soldered to the prototyping shield, and crimped pin connectors were used for connection to the electronic components.

### 2.4. External Device Interconnection

External connections to the EMCS were made using RJ45 ethernet jacks and commonly available ethernet cables. An advantage of using RJ45 cables for device interconnection is they cannot be plugged in backwards, so the risk of incorrectly connected components and short circuits was avoided. The pin configuration was set up such that connecting the wrong device to a jack would not result in a short circuit or damage to the Arduino microcontroller. This was accomplished by keeping the same voltage or signal levels from left to right across jack pins. Every other pin was connected to ground to prevent electrical signal interference in data cables.

The base configuration of the EMCS contained three RJ45 jacks. A fourth RJ45 jack was added in a housing extension fastened between the main enclosure and bottom lid. An advantage of this design is the ability to easily swap or reconfigure an RJ45 jack for another purpose by connecting a different female connector with different wires.

#### 2.4.1. Air Speed Sensor

**Figure 3:**
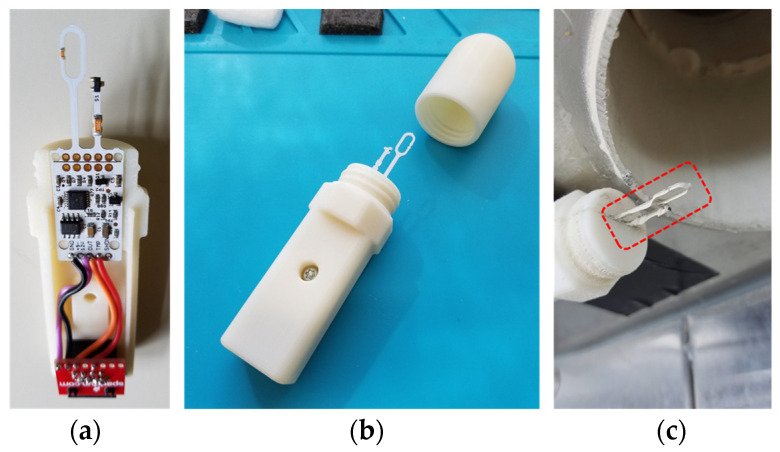
Photos of (**a**) WindSensor mounted in 3D-printed housing, (**b**) assembled housing with protective cap unscrewed, and (**c**) housing taped to ceiling with sensing tip inserted into airstream below ceiling exhaust pipe.

Air speed (m/s or ft/min) was measured using a WindSensor (Rev.P, Modern Device, Providence, RI), which is a low-cost thermal-based anemometer with performance that compares favorably with instruments costing many times as much [38]. The WindSensor required 9–12 VDC, was powered using 12 V, and returned a 0–3.3 V analog voltage correlating to an air speed of 0–241 km/h (0–150 mph). The sensor was housed externally in a custom designed 3D-printed ABS plastic enclosure (Figure 3a). The housing had a simple split design that exposes a heated thermistor to moving air and included a threaded cap to protect the tip during transportation or storage (Figure 3b). The other end of the housing contained an RJ45 jack for connection to the microcontroller. The housing was taped to the ceiling with the heated sensing element inserted into the air stream below an exhaust duct (Figure 3c).

#### 2.4.2. Dust Sensor

**Figure 4:**
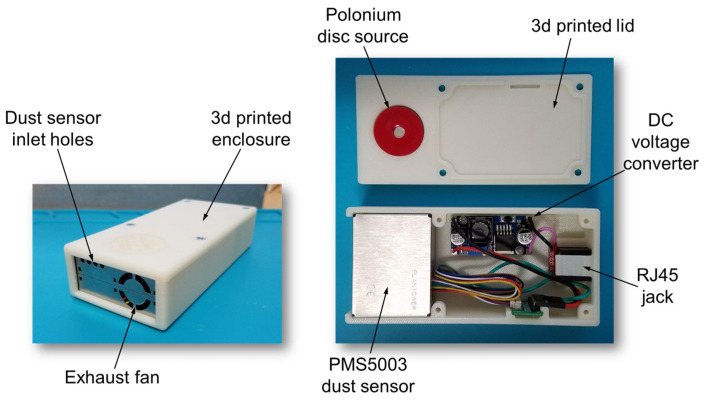
Dust sensor in 3D-printed housing.

Dust concentration was measured using a Plantower PMS5003 laser particle counter (Plantower, Beijing, China). The dust sensor was housed externally in a custom 3D-printed ABS plastic enclosure [39] shown in Figure 4. The custom dust sensor enclosure contained a DC-DC voltage converter to convert supplied 12 V to 5 VDC, and an RJ45 jack (SparkFun Electronics, Niwot, CO, USA) was used to connect the dust sensor to the microcontroller using an ethernet cable. A recess in the housing lid held a Polonium-210 disk source (United Nuclear Scientific, Klamath Falls, OR, USA) in contact with the top of the dust sensor to eliminate static and prevent buildup of dust particles on plastic surfaces inside the sensor.

#### 2.4.3. Dust Generator

Airborne dust was dispersed using a custom-built dust generator [39] controlled by the EMCS based on real-time dust particle counts from the PMS5003 laser particle counter. One RJ45 jack in the environment monitor housing was used to connect to the dust generator for control using an ethernet cable.

#### 2.4.4. Ammonia Generator

Airborne ammonia gas was supplied using a custom-built ammonia generator [35] controlled by the EMCS based on real-time ammonia gas concentration measurements from the MQ-137 ammonia gas sensor. One RJ45 jack mounted in a housing extension was used to connect to the ammonia generator via an ethernet cable.

#### 2.4.5. Signal Conditioning

A cumulative moving average (CMA) filter [40] was used to smooth real-time measured MQ-137 ammonia sensor reading fluctuations for better control over gas output from the connected ammonia generator:

CMA_n+1_ = (X_n+1_ + n × CMA_n_)/(n + 1) (1)

where n is the period length, X_n+1_ is the next measured value, and CMA_n_ is the cumulative moving average of the last n measurements. The larger the period length, n, the quicker the controller will respond to changes in measured concentrations. This method is useful for microcontrollers with small amounts of program memory because it does not require storage of the past n values, only storage of the calculated CMA, which gets updated as each X_n+1_ gets measured. This also means fewer lines of program code are needed to implement the filter.

### 2.5. Software Integration

The Arduino setup function initializes the real-time clock, microsd card module, and connected digital sensors to make sure they can communicate with the microcontroller. Then, for safety, commands get sent to the dust and ammonia generators to turn them off to prevent accidental operation after a power loss. The main loop then reads values from all connected sensors every 5 s (5000 ms) and logs them to the SD card. The ammonia and dust generators then get controlled based on measured NH_3_ and PM10 concentrations.

#### 2.5.1. Ammonia Speed Control Algorithm

Ammonia generator control was based on measurements from the MQ-137 sensor. Because ammonia concentration measurements fluctuations cause frequent starting and stopping, a CMA filter was used to smooth the signal for steady operation. The desired ammonia setpoint concentration for the experiment was 50 ppm. When the measured ammonia concentration fell below the desired setpoint, the ammonia generator speed was varied in response to the real-time measured concentration using Algorithm 1.
**Algorithm 1**: Ammonia Generator Speed Control Algorithm// define CMA period length period = 5.0; // 30 reacts slower for 2% ammonia liquid        // 5 reacts faster for 10% ammonia liquid // apply a CMA filter to the MQ-137 sensor readings filteredPpm1 = (MQ137_ppm + period * filteredPpm0)/(period + 1);  filteredPpm0 = filteredPpm1; // change ammonia generator output level based on measured concentration if(filteredPpm1 >= 50.5) { NH3_speed = 0; }//Turn NH3 generator OFF else if(filteredPpm1 >= 48.5 && filteredPpm1 < 50.5) { NH3_speed = 2; } else if(filteredPpm1 >= 46.5 && filteredPpm1 < 48.5) { NH3_speed = 3; } else if(filteredPpm1 >= 44.5 && filteredPpm1 < 46.5) { NH3_speed = 4; } else if(filteredPpm1 >= 42.5 && filteredPpm1 < 44.5) { NH3_speed = 5; } else if(filteredPpm1 >= 40.5 && filteredPpm1 < 42.5) { NH3_speed = 6; } else if(filteredPpm1 >= 38.5 && filteredPpm1 < 40.5) { NH3_speed = 7; } else if(filteredPpm1 >= 36.5 && filteredPpm1 < 38.5) { NH3_speed = 8; } else if(filteredPpm1 < 36.5) { NH3_speed = 8; }

#### 2.5.2. Dust Control System Algorithm

Dust generator control was based on measurements from the PMS5003 sensor using PM10 as a control variable because it did not fluctuate as rapidly during preliminary testing as the other values measured by the PMS5003 sensor, leading to less frequent starts and stops for smoother operation. The desired PM10 setpoint concentration of airborne dust used for the experiment was 50 µg/m^3^. When the measured PM10 concentration fell below the desired setpoint, the dust generator was operated using a duty cycle of 1 out of every 5 s (1 s ON, 4 s OFF) using Algorithm 2.
**Algorithm 2**: Dust Generator Control Algorithm// define dust generator control variables Dcontrol = 50;    // desired PM10 setpoint to maintain dustrun = 1000; // duration to run dust motor, milliseconds  // check to see if dust generator is operating, stop if run duration is reached if(Dust_speed == 1 && (millis() - duststart) > (dustrun))  {    stopMotor();      // sends signal to stop dust generator motor   Dust_speed = 0; // an indicator variable logged to show if motor is on or off } // check to see if PM10 concentration is above desired setpoint if (PM10 < Dcontrol)  {    duststart = millis();    turnMotor(255);  // turn the dust generator motor at full speed    Dust_speed = 1; } else stopMotor();

### 2.6. Data Communication

An on-site desktop PC was located in a laboratory across the hallway from the four controlled environment chambers. The PC used the Windows 10 operating system and had a wired internet connection. One EMCS was deployed in each chamber, and each was connected to the desktop PC using long USB cables. This made it simple to access each Arduino microcontroller from the PC to view live measurements via the serial monitor and to make program changes if needed. Four separate instances of the Arduino IDE were run simultaneously within Windows 10, and the active serial port in each instance was changed so each program window accessed a different EMCS in each chamber. A serial monitor window was opened for each instance so measurements from all four chambers could be viewed on-screen at the same time. Each EMCS printed an identifier at the end of each line of data to make it easy to determine which chamber was being viewed in the serial monitor window.

### 2.7. Power Supply and External Enclosure

**Figure 5:**
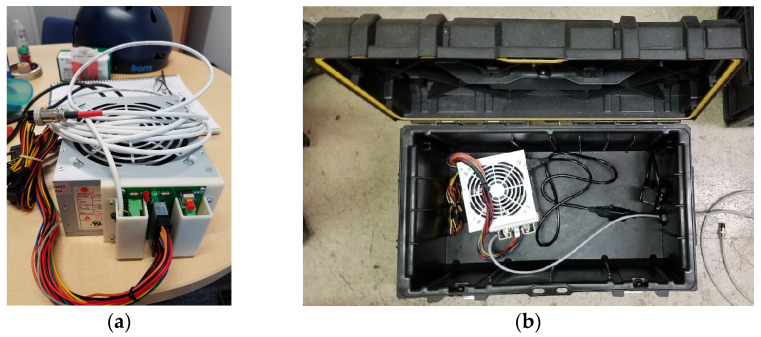
(**a**) ATX power supply, breakout board, and 3D-printed mounting adapter; (**b**) power supply inside a toolbox with power cables passed through cable glands.

The Arduino MEGA 2560 microcontroller could supply 40 mA per I/O pin, up to a total of 200 mA for connected devices. Devices with low power requirements were powered directly from the microcontroller, but devices requiring high current were powered by an external 500-watt ATX power supply (CoolMax model I-500, CoolMax Technology Inc., Taipei, Taiwan). A 24/20-pin ATX DC Power Supply Breakout Board Module (Electronics-Salon P/N MD-D1188-1, CZH-LABS, Shenzhen, China) was mounted to the power supply using a custom 3D-printed mounting adapter (Figure 5a). The ATX breakout module was used to connect a 22 gauge 4-wire shielded power cable supplying 12 V, 5 V, 3.3 V, and GND connections to the datalogger housing socket using a keyed 16 mm 4-pin jack and plug to prevent the connector from being connected incorrectly. The supply voltages were monitored by connecting them to the prototyping shield using resistors as a voltage divider and analogRead measurements for troubleshooting purposes. A LM2596 DC-DC step-down converter (HiLetGo, Shenzhen, China) was used to get 7.5 V from the 12 V line and was used to power the Arduino MEGA 2560 microcontroller and the K30 CO_2_ sensor. To compensate for voltage drop, all externally connected sensors were powered using 12 VDC over ethernet cables, and voltage converters inside the external sensor enclosures were adjusted to output 5.2VDC.

### 2.8. Rugged External Case

The ATX power supply was housed in a rugged, waterproof toolbox (ToughSystem 22 in. toolbox, DeWalt, Towson, MD, USA) which had space to hold all components, cables, and accessories during transportation and a large internal air volume to prevent the power supply from overheating. Two openings were added to the toolbox for power cables: one for a weatherproof cable gland used for the 24 gauge 4-conductor power supply cable to the EMCS, and a 120V RV/camper bulkhead was used to supply 120VAC to the ATX supply. The toolbox was also used to store and transport the EMCS, air speed sensor, dust sensor, and power and data cables. The toolboxes could be stacked and locked together to a wheeled base (ToughSystem 22 in. mobile toolbox, DeWalt, Towson, MD, USA; used for one EMCS). Figure 5b shows the ATX power supply inside a toolbox with power cables passed through cable glands.

### 2.9. Case Study: Six-Week Broiler Chicken Airborne Contaminant Study

Four environment measurement and control systems were deployed in four controlled environment chambers at the Poultry Education and Research Center (PERC) at the Pennsylvania State University with different treatments for a six-week broiler chicken health experiment to evaluate the physiological effects of exposure to controlled levels of ammonia gas and airborne dust [4]. Detailed methods and results are found in [4], but a brief outline of study is provided here.

The environment-control chambers were constructed by Thermolinear (Cincinnati, OH, USA) and had dimensions of 3.7 m × 4.3 m × 2.4 m (12 ft × 14 ft × 8 ft) with stainless steel walls, flooring, and ceilings. Each chamber contained a continuously operated exhaust fan duct located in one corner of the ceiling to exchange indoor air with fresh outdoor air drawn into chamber inlets at an air flow rate of 0.06 m^3^/s (130 cfm). Temperature, humidity, and lighting in each chamber were controlled by a Honeywell UDC-5000 controller (Honeywell, Charlotte, NC, USA). Temperature could be controlled between 4 °C and 40 °C (±0.5 °C), and humidity (at upper and lower temperature limits) from 60 to 90% at 4 °C, and from 25 to 80% at 40 °C. Humidity was added to the chamber by a Nortec EL-50 steam humidifier (Nortec Humidity Ltd., Ottawa, ON, Canada). An air handling unit recirculated 1.5 m^3^/s (3000 cfm) of air in the chamber to maintain temperature and humidity settings. The chamber dehumidifiers were not operable during the experimental timeframe. Daily chamber maintenance was performed between 8:00 and 10:00 a.m. and consisted of refilling the ammonia generator reservoir and dust generator hopper, cleaning ventilation air filters, wiping accumulated dust from the walls and floor, and cleaning any settled dust from the outside of instrument housings and vacuuming sensor openings to prevent blockage from large airborne particles or feathers. Chamber temperature was set to 33.3 °C on day 1 and progressively reduced to 23.3 °C on day 42. The lighting photoperiod was 22 h with an intensity of 30 lux followed by a 2 h dark period. The treatments used for each chamber were:

- Chamber P1: Experimental control. EMCS #1 only used to record environmental variables.
- Chamber P2: Birds exposed to airborne dust. EMCS #2 recorded environmental variables and controlled a dust aerosol generator [39] based on dust particle counts to maintain a steady airborne dust concentration.
- Chamber P3: Birds exposed to both ammonia and airborne dust. EMCS #3 recorded environmental variables and controlled both an ammonia generator [35] and a dust generator [39] to maintain steady concentrations of ammonia gas and airborne dust.
- Chamber P4: Birds exposed to ammonia gas. EMCS #4 recorded environmental variables and controlled an ultrasonic ammonia generator [35] based on ammonia gas sensor feedback to maintain a steady ammonia gas level of 50 ppm.

The same base Arduino program was used for all four EMCS microcontrollers, but dust and ammonia generator control subroutines were used in the EMCS units that controlled ammonia gas and/or dust aerosol generators. The system was remotely monitored multiple times each day. The Arduino IDE serial monitor windows could not be left running unattended due to buffer capacity issues that would cause the serial monitor window to freeze when allocated memory ran out. So, the daily operating procedure was to connect to the lab PC using Remote Desktop Protocol (RDP), then open the serial monitor for each instance of the Arduino IDE that was running, inspect the data streams, then close the serial monitor windows before logging out of the remote session. This facilitated remote viewing and code changes at any time of day from any location using a computer with an internet connection.

Room calibration measurements were made on-site by a student who cared for the animals in the morning and late afternoon. During each visit to the chambers, ammonia concentration was spot checked using handheld reference instruments. A handheld multi-gas meter (Model MX6, Industrial Scientific, Pittsburgh, PA, USA) was placed next to the EMCS in each chamber for several minutes to compare readings to MQ-137 ammonia concentration measurements. If the MQ-137 sensor readings differed by more than 5%, remote adjustments were made to calibration values for the ammonia sensors over the Remote Desktop connection.

## 3. Results

Four environment measurement and control systems were successfully operated continuously for a six-week broiler health study [4]. Each EMCS recorded approximately 100,000 rows of data per week with measured temperature, relative humidity, luminosity, carbon dioxide concentration, ammonia gas concentration, dust concentration, and air speed. The ammonia gas and dust generators were automatically controlled based on real-time measurements with a high level of accuracy [35,39]. No technical difficulties were experienced during the study. The EMCS proved to be stable, even in the presence of high concentrations of ammonia gas and dust. The only daily maintenance required was to remove accumulated dust from the surfaces of enclosure lids and exposed sensors. The total cost for components to build one EMCS was less than $500 USD (including shipping costs and ABS 3D printer filament).

### 3.1. Control of Ammonia Concentration

During the six-week experiment, two ammonia generators were deployed using 10% ammonia liquid in two separate controlled environment chambers. The EMCS automatically operated the ammonia generators and maintained an average ammonia concentration of 45.63 ± 4.95 ppm in Chamber P3 and 46.42 ± 3.81 ppm in Chamber P4 for the entire duration of the experiment.

### 3.2. Control of Dust Concentration

During setup for the six-week experiment, it was observed that the dust generator in chamber P3 (dust + ammonia) was not dispersing as much dust as the generator in chamber P2 (dust only). This was because the PMS5003 sensor in chamber P3 was reading false high levels of particulate matter because the ammonia generator in the chamber emitted a fine mist during operation that was detected as particulate matter by the PMS5003 sensor. As a workaround, the height of dust remaining in the dust generator hoppers was measured each day in Chambers P2 (dust only) and P3 (dust + ammonia), and minor adjustments to the control level for the dust generator in chamber P3 were made if required to ensure the same daily volume of dust was being dispersed as in chamber P2. Additionally, plastic cards measuring 10 cm × 10 cm (100 cm^2^) were placed inside each chamber to collect settled dust for 24 h. The cards were then weighed each day (to the nearest milligram), and the weight of settled dust was recorded as a measure of airborne dust uniformity between the two chambers.

Over the six-week experiment, the average daily weight of settled dust on the card in chamber P2 (dust only) was 0.117 g/100 cm^2^, and the average daily weight in the chamber P3 (dust + ammonia) was 0.162 g/100 cm^2^. The average PM10 concentration measured by the PMS5003 sensor in chamber P2 (dust only) over six weeks was 54.92 ± 6.42 µg/m^3^, and the average in chamber P3 (dust + ammonia) was 66.78 ± 20.07 µg/m^3^.

### 3.3. Measured Temperature and Relative Humidity

**Figure 6:**
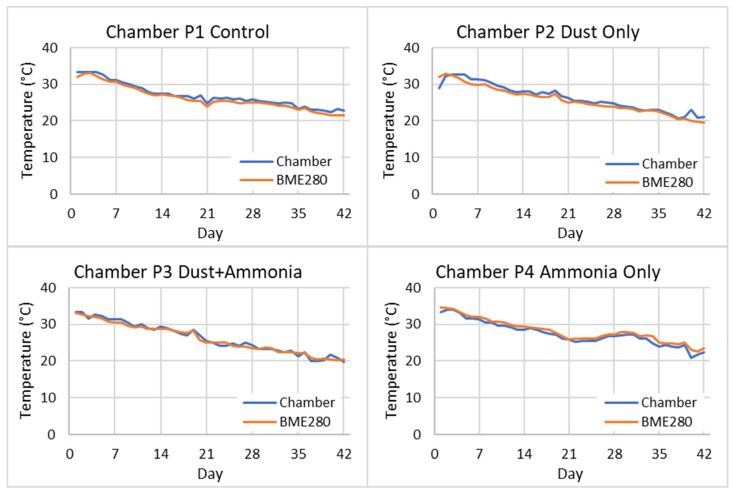
Recorded daily temperatures from chamber controllers and BME280 sensors.

Since the EMCS units were used to measure but not to control chamber temperature and relative humidity, no calibration adjustments were made for the BME280 sensors. Figure 6 shows the recorded daily temperatures recorded from the chamber controllers compared to the BME280 sensor readings in each chamber for all six weeks of the study. Table 2 lists comparison statistics between BME280 and chamber controller temperature readings over the six-week study. The uncalibrated BME280 temperature measurements had a mean absolute error (MAE) within 0.90 °C (± 0.67 °C) of the displayed chamber controller temperature readings and were highly correlated with Pearson’s *r*(40) ≥ 0.97, *p* < 0.001 for all four chambers.

**Figure 7:**
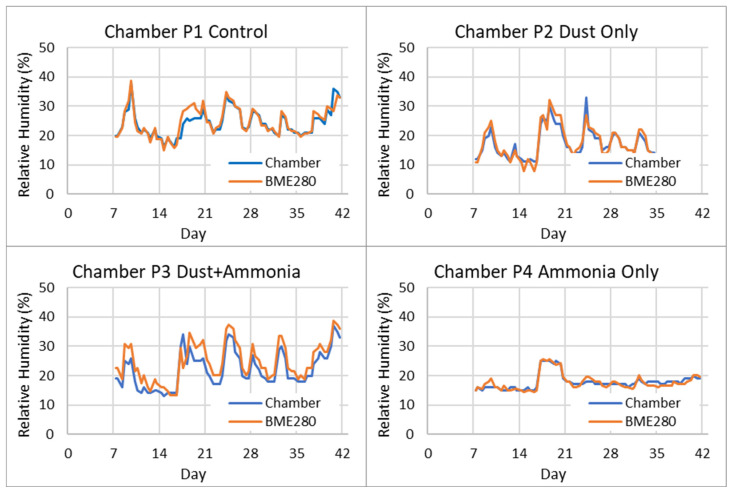
Recorded daily humidity from chamber controllers and calibration-adjusted BME280 sensor readings.

BME280 humidity readings during the six-week experiment were logged for comparison to the displayed chamber controller values. Chamber humidity sensors were found to be faulty and replaced during the first week of the experiment, so only values from weeks 2 to 6 were used for comparison. Linear regression of BME280 versus chamber controller humidity was used to calculate calibration correction coefficients (slope and offset) shown in Table 3. Figure 7 shows daily humidity recorded from the chamber controllers compared to the corrected BME280 sensor readings in each chamber for the last five weeks of the study, and Table 4 lists comparison statistics between BME280 and chamber controller humidity readings. The corrected BME280 humidity measurements had a mean absolute error (MAE) within 3.17% (± 1.76%) of the displayed chamber controller humidity readings and were highly correlated with Pearson’s *r*(33) ≥ 0.93, *p* < 0.001 for all four chambers.

### 3.4. Case Study Observations

There were three instances during the six-week study when conditions inside the controlled environment chambers required attention when issues were detected remotely when monitoring sensor readings from the EMCS. Daily monitoring enabled detection of many other events remotely, and the author was able to call the student worker when conditions seemed different. A few times, the student spilled water on the floor while refilling the drinkers, and the relative humidity increased noticeably compared to the other chambers. It was also detectable when the student was cleaning the chamber air filters because the dust concentration would rise sharply compared to normal background or generated levels of dust. The luminosity sensor made it possible to remotely detect issues in two chambers where incorrectly configured lighting timers failed to turn off chamber lights at night or caused a regularly occurring lights-off period of one hour every day. These types of issues, if undetected, could lead to unexplainable differences in experimental results because many farm animals are sensitive to photoperiod.

The air speed sensor was simply used as an indicator to detect if the chamber exhaust velocity varied during experiments, not for highly precise analytical purposes. Still, it returned reasonable values around 7.6 m/s (1490 ft/min) when monitoring air speed at the chamber exhaust pipe compared to expected values calculated from the rated exhaust fan flow rate of 0.06 m^3^/s (130 cfm) and exhaust pipe inside diameter of 101.6 mm (4 in.). It was a useful indicator of when chamber air filters required cleaning because measured air speed would steadily decrease as dust accumulated on the air filters. Detection of empty ammonia generator reservoirs or dust generator hoppers was also possible because measured ammonia or dust concentrations would decrease suddenly and the generators would operate at full speed to try to maintain set concentrations.

## 4. Discussion

One of the main advantages of using the Arduino MEGA microcontroller is the capability to expand the system as needed. With extensive I/O and flash memory capacity, sensors and controlled equipment can be added or removed with minimal hardware redesign. Code can easily be modified to reconfigure the system for different tasks and can incorporate complex conditional logic to trigger events when a threshold is reached. Another important aspect of an integrated sensor network is to log data from multiple sensors to one log file with the same timestamp to reduce errors [12]. If multiple loggers are used, even if internal clocks are closely synchronized, it is possible for events to be logged at different times, making subsequent data analysis more difficult since events need to be grouped or lined up in time.

Another advantage of using a programmable microcontroller is the ability to apply custom real-time signal filtering in software. Especially when measurements are made at a high sampling rate, signal conditioning may be needed to smooth measured values. An easy way of achieving this is to apply a cumulative moving average (CMA) filter. The length of the period used for the moving average filter can be adjusted, with a longer period resulting in a smoother signal that reacts more slowly to changes in measured concentration. A shorter period allows for quicker reaction to changes in ammonia concentration but results in a less steady signal.

With so many connected sensors and integrated controls, it was possible to easily detect failing components and write code to react and send a notification, sound an alarm, or alter operating parameters to adjust output from controlled equipment. For example, the air speed sensor was very useful for detecting changes in the chamber ventilation system due to dirty air filters and differences in fan controller operation from room to room. A low air speed measurement by itself could indicate a dirty or faulty sensor, or issues such as a clogged air filter. When combined with the luminosity sensor, a reduction in measured air speed with no change in measured light levels would be an indication that the chamber exhaust fan required maintenance or was failing.

### 4.1. Software Integration Challenges

At least for the Arduino integrated development environment (IDE) which was used to develop code executed by the microcontroller, the experience of the authors was that sometimes software libraries for different sensors would cause problems that prevented code from working as intended. In many cases, it was necessary to add one component at a time to the microcontroller, then find a software library that worked adequately and allowed access to the component. Therefore, once a working combination of connected components and software libraries is found, they should not get updated when new versions are available unless the update includes additional functionality that is specifically needed, because in some cases the update may cause problems with other included libraries or components. During development of the EMCS described in this paper, the same version of the Arduino IDE (1.8.1) and the included software libraries for each connected component were always used. Attempting to run the developed code with newer versions of software libraries typically resulted in compilation errors.

#### 4.1.1. Ammonia Generator Control Tuning

Ammonia concentration rises more rapidly when stronger concentration liquid ammonia is used in the ammonia generator. To compensate for different strength ammonia liquid, the CMA period length can be changed along with control levels used to switch ammonia generator output speeds.

A moving average filter period of 30 readings was used in the control system with 2% ammonia liquid to act as a signal damper for the rapidly fluctuating ammonia measurement signal. Ammonia generator output speed was controlled using an increment of 0.25 ppm. The generator was started at the highest speed until the measured ammonia concentration reached 48.75 ppm, then speed was decreased one step for every 0.25 ppm increase in concentration. The ammonia generator was stopped if the measured concentration reached 50.5 ppm.

A smaller moving average filter period was used in the control system to allow the single generator to react more quickly to changes in ammonia concentration. The filter period was changed from 30 readings used for 2% ammonia liquid to 5 for 10% ammonia liquid. The control levels used to change speeds also had to be adjusted from 0.25 to 2.0 ppm increments, starting at full speed until the concentration reached 36.5 ppm, then decreasing by one step for every 2.0 ppm increase and stopping at 50.5 ppm. Table 5 lists ammonia generator output speeds for each control level with tuning parameters for use with 2% and 10% strength liquid ammonia.

#### 4.1.2. Dust Generator Control Tuning

During early testing, the dust generators were initially operated using pulse width modulation (PWM) so the dust conveyor belt speed could be varied to vary dust output. However, the dust conveyor gearmotor would not start if the PWM speed was less than 150, which is not even half of the full speed of 255. So, the dust generator was operated at full speed for the Arduino loop duration when the control level fell below the desired setpoint. This also caused a problem, because the large volume of dust dispersed during 5 s of full speed operation quickly overshot the setpoint concentration and was slow to fall below the control level, resulting in short bursts of high airborne dust concentration followed by long idle periods. The solution that worked best was to vary the dust generator operating duty cycle in the Arduino control algorithm. It was determined that a duty cycle of 1 out of every 5 s resulted in very stable levels of airborne dust and more uniform operation of the dust generator.

### 4.2. Observations from the Six-Week Broiler Health Study

The developed EMCS improved the way experiments were conducted by enabling problems to be detected early for correction. A technician fed and watered the birds multiple times each day, performed daily cleaning tasks, and communicated any monitoring concerns twice daily. The ability to connect to the EMCS remotely from any Windows PC was a powerful tool that allowed issues to be addressed very quickly and eliminated unnecessary travel to the research facilities. Similar benefits can be realized in commercial facilities, where the cost of not noticing a problem could be very high due to the larger scale of operations and distance between multiple facilities.

### 4.3. Component Issues and Workarounds

#### 4.3.1. Real-Time Clock Drift

There are different versions of DS3231 real-time clock modules available for purchase. Initial modules tested included the DS3231M chip [41], which uses a temperature compensated MEMS resonator and has an advertised time accuracy of ±5 ppm, but in testing it resulted in unacceptable amounts of time drift of several minutes every week. These were replaced with RTC modules that included the DS3231SN chip [37], which uses a 32 kHz oscillator with an accuracy of ±2 ppm that maintained the correct time and date with no noticeable drift over the duration of the study.

#### 4.3.2. Temperature and Relative Humidity Sensor

During development of the EMCS, a DHT22 sensor was tested for measurement of temperature and relative humidity. The DHT22 sensor was initially mounted inside the 3D-printed EMCS housing, but during testing, it was observed the DHT22 was artificially heated by other electronic components inside the enclosure, such as the voltage regulator on the microcontroller, which caused temperature readings to be too high. The solution was to mount the DHT22 sensor in a 3D-printed enclosure and connect it externally using a short length of instrument cable. During further testing, the DHT22 sensor failed soon after use in the continuous presence of 50 ppm of ammonia gas. The failed sensor was replaced with a BME280 temperature/humidity/pressure sensor which performed well without failure during six weeks of continuous use in the presence of 50 ppm of ammonia gas. Relative humidity readings from both the DHT-22 and BME280 sensors were offset by as much as ±10% compared to reference instruments in some cases, but it was determined they only needed an offset adjustment in the program code, after which they returned correct measurements.

### 4.4. Dust Sensor Observations

Over the six-week experiment, there were no PMS5003 sensor issues related to clogging or drift under heavy dust loads. Daily chamber maintenance included gently wiping dust sensor openings with a soft bristle brush to remove any coarse particles like feathers to prevent occlusion of the air sampling openings. The 3D-printed sensor enclosure also included a Polonium-210 disk source that emitted alpha particles to eliminate static and prevent dust from adhering to plastic sensor passages, and that may have also prevented clogging. Polonium-210 has a relatively short half-life of 138 days [42], however, so the disk source may need to be replaced as often as every 18 months to remain effective.

The PMS5003 sensors were disassembled for inspection at the conclusion of the six-week experiment, and no significant buildup of dust was observed in the flow passages. However, there was a visible film on the collector of one of the PMS5003 sensors in chamber P3 where both dust and ammonia were generated, probably due to very fine dust in addition to mist from the ammonia generator. The film did not negatively impact sensing compared to the other three PMS5003 sensors used in the experiment, and it was easily wiped clean with a soft cloth. We recommend periodic inspection and cleaning of optical sensors if issues are suspected during operation in very dusty and humid environments.

### 4.5. Ease of Adding or Changing Connected Sensors

The transition from the DHT22 to a BME280 temperature/humidity sensor illustrates the practical benefits of the modular design of the EMCS. Initially, the DHT22 sensor was wired to digital pin 2 and accessed via the DHT library. To replace it with the BME280, we simply connected the sensor to the Arduino I2C bus (SDA/SCL pins) and updated the code to include the BME280 library. This required only a four-wire jumper between the BME280 and the MEGAshield I2C header, eliminating the need to remove the prototyping shield or solder additional leads. This example demonstrates that software and hardware can be reconfigured rapidly and enables the same datalogging framework to accommodate a wide range of commercially available sensors without costly hardware redesign.

Similarly, a Shinyei PPD42 dust sensor [43] was initially tested for suitability in the EMCS by connecting its digital outputs to pins 3 and 4 on the MEGAshield. Replacing the PPD42 with the more reliable Plantower PMS5003 sensor only required addition of a single jumper from the sensor TX to the MEGAshield RX3 pin (hardware serial port 3) to enable UART communication. The code was modified to include a subroutine to read serial data from the PMS5003 sensor. Since the RJ45 jacks in the EMCS housing get connected to the MEGAshield using single-row male pin headers, it is easy to make changes to the pin configuration for different sensors or controlled equipment. This illustrates platform modularity, allowing seamless hardware and corresponding software changes without extensive rewiring.

Furthermore, changing what sensor data gets measured and logged is a simple matter of coding. For instance, the TSL2561 luminosity sensor [44] can output full spectrum, infrared, and visible light levels (visible = full spectrum − infrared). A minimal code modification allows logging of only the visible channel, or to append the full spectrum and infrared readings if required. Likewise, the BME280 sensor measures temperature, relative humidity, and barometric pressure; by making appropriate coding changes, the EMCS can switch from a two-parameter configuration (temperature + humidity) to a three-parameter one that also records pressure without any change to the wiring or board layout. This flexibility means new data fields can be introduced or omitted as desired through simple programming changes, facilitating rapid prototyping and iterative refinement of the environmental monitoring pipeline as technology changes.

### 4.6. Data Processing and Microcontroller Code Advantages

Each time the Serial Monitor was opened, a new header row was written to the data file with program filename and the date and time the program was last compiled, which was useful for tracking program changes in case a new sensor was added, changed, or additional measurements or variables were logged to the data file. Post-processing of CSV data files involved running an Excel macro to remove all extra header rows. Also, the Timestamp (milliseconds) variable in the first column of data was reset each time the Serial Monitor was opened or the program was reset, which would also happen in case of a power failure. By plotting a graph of Timestamp value versus Time, it was easy to see when and how many times the program was reset or the serial monitor was opened.

If multiple dataloggers will be deployed for the same study, and different sensors or devices will be monitored or controlled, the data logs may contain different numbers of columns, making post-processing and data analysis tedious. It may be worth creating a list of all measured variables, using the same data header row for all dataloggers, then simply writing blank values (just a comma instead of a value followed by a comma) to the data log so the files will have the same number of columns when opened in Excel.

### 4.7. Considerations for Portability

If control of external devices is not required, the EMCS can easily be used as a simple datalogger and powered using batteries. For a truly portable environment monitor, some of the selected sensors may be omitted or replaced with low-power consumption devices. For example, the MQ-137 ammonia sensor consumes ~200 mA of power at 5 VDC, and since the sensor takes several minutes for the metallic oxide semiconductor to reach operating temperature and must remain heated during sensing, deactivating the sensor between measurements is not an option for continuous monitoring. A rechargeable 5 V 8 Ah battery pack will fit inside the EMCS enclosure, and under perfect operating conditions could power one MQ-137 sensor alone for up to 40 h, but this does not account for the Arduino microcontroller or other connected sensors. If longer runtime is needed, lower-power sensors may need to be considered.

### 4.8. Possible Connection to an IoT/Cloud Platform

Although it was not utilized during the six-week experiment, the included ESP8266 Wi-Fi module makes it possible to connect the EMCS to an IoT cloud platform, such as the native Arduino IoT Cloud, ThingSpeak, If This Then That (IFTTT), and many others. This would enable users to create a dashboard to conveniently display desired sensor measurements and make simple changes to controls from any device using a web browser.

## 5. Conclusions

A highly integrated, custom-built all-in-one environment measurement and control system (EMCS) was developed for animal health research studies. The system was deployed in four controlled environment chambers for a six-week broiler health study and recorded luminosity, carbon dioxide and ammonia gas concentrations, dust particle counts, air speed, temperature, and humidity while controlling ammonia and dust generators to maintain steady concentrations of ammonia gas and dust. The EMCS was robust and provided stable operation in harsh environments similar to animal barns containing elevated concentrations of ammonia gas and airborne dust. The developed system improved the way we conducted experiments and enabled rapid response to issues while greatly reducing the need to visit the site, since adjustments could be made remotely and quickly. The developed environment measurement and control system is inexpensive, easy to use, modular, and adaptable to a wide range of usage scenarios.

## Figures and Tables

**Table 1:** List of measured variables printed to serial monitor window and logged to storage card.

Component	Variable	Units
Arduino	Timestamp	milliseconds
DS3231 RTC	Date and Time	mm/dd/yyyy and hh:mm:ss
TSL2561	Light level	Lux
BME280	Temperature, humidity	°C, RH %
MQ-137	Ammonia gas concentration	ppm
K30	Carbon dioxide gas concentration	ppm
WindSensor	Air speed	ft/min
PMS5003	PM10	µg/m^3^
Supply Voltage	3.3 V, 5 V, 7.5 V, 12 V voltage	volts

**Table 2:** Statistics for comparison of BME280 and Chamber Controller temperature values.

Statistic	Temperature °C			
	Chamber #1	Chamber #2	Chamber #3	Chamber #4
MAE *	0.73	0.90	0.53	0.74
SD	0.38	0.67	0.33	0.42
Pearson’s r ^†^	0.99	0.97	0.99	0.99

* Mean absolute error = $\sum_{n}^{1}|($BME280 value—Chamber controller value)|/n; ^†^ *p* < 0.001 for all four chambers.

**Table 3:** Linear regression coefficients for BME280 humidity calibration.

Linear Regression Coefficients	Chamber #1 BME280	Chamber #2 BME280	Chamber #3 BME280	Chamber #4 BME280
m	1.05	0.99	0.75	1.68
b	−6.34	2.13	8.01	−11.30
R^2^	0.86	0.86	0.88	0.85

**Table 4:** Statistics for comparison of BME280 and Chamber Controller humidity values.

Statistic	Relative Humidity (%)			
	Chamber #1	Chamber #2	Chamber #3	Chamber #4
MAE *	1.22	1.34	3.17	0.75
SD	1.49	1.17	1.76	0.59
Pearson’s r ^†^	0.93	0.95	0.94	0.94

* Mean absolute error = $\sum_{n}^{1}|($BME280 value—Chamber controller value)|/n; ^†^ *p* < 0.001 for all four chambers.

**Table 5:** Ammonia generator output speed, control levels, and tuning parameters for different strength ammonia liquid.

Output Speed	Control Level (ppm)	
0 (off)	ppm ≥ 50.5	ppm ≥ 50.5
1	50.5 > ppm ≥ 50.25	Not used
2	50.25 > ppm ≥ 50.0	50.5 > ppm ≥ 48.5
3	50.0 > ppm ≥ 49.75	48.5 > ppm ≥ 46.5
4	49.75 > ppm ≥ 49.5	46.5 > ppm ≥ 44.5
5	49.5 > ppm ≥ 49.25	44.5 > ppm ≥ 42.5
6	49.25 > ppm ≥ 49.0	42.5 > ppm ≥ 40.5
7	49.0 > ppm ≥ 48.75	40.5 > ppm ≥ 38.5
8 (full)	48.75 > ppm	38.5 > ppm
Ammonia Strength	2%	10%
Filter Constant	30	5

## Data Availability

The raw data supporting the conclusions of this article will be made available by the authors on request.

## Appendix A

Table A1 is a list of parts and electronic components used to build the environment measurement and control system.

**Table A1:** Parts list for environment measurement and control system.

Quantity	Component
1	Arduino MEGA 2560
1	MEGAshield, for Arduino Mega 2560 R3
1	Wind Sensor Rev. P
1	PMS5003 Dust Sensor with cable
1	Polonium-210 Disk Source
1	TSL2561 Luminosity Sensor (0.1–40,000+ Lux)
1	LM2596 DC-DC buck converter
1	MQ-137 Ammonia Sensor 5–500 ppm
1	K30 1% NDIR Carbon dioxide sensor 0–10,000 ppm
1	DS3231 AT24C32 IIC precision RTC module w/battery
1	BME280 Temperature, humidity, and pressure module
1	ESP8266-01S WiFi Microcontroller for Arduino
1	24-pin ATX Power Supply Breakout Board
1	500 W ATX 12 V Power Supply
1	MicroSD Card 16 GB Class 10
1	MicroSD Card Adapter
1	0.96 inch yellow blue I2C OLED LCD module
1	Waterproof aviation connector 16 mm 4 pin
4	RJ45 breakout board
1	Shallow clear polycarbonate cover for enclosure
**Additional items needed for assembly**	
	2.54 mm pitch JST-SM crimp connectors
	22 awg stranded silicone wires
	Metal film resistors 1% 1/4 W through hole
	Cat5e 15ft ethernet patch cables
	3:1 dual wall adhesive heat shrink tubing kit
	Thread forming screws for plastic
